# Synergistic Pt-WO_3_ Dual Active Sites to Boost Hydrogen Production from Ammonia Borane

**DOI:** 10.1016/j.isci.2020.100922

**Published:** 2020-02-19

**Authors:** Wenyao Chen, Wenzhao Fu, Gang Qian, Bingsen Zhang, De Chen, Xuezhi Duan, Xinggui Zhou

**Affiliations:** 1 State Key Laboratory of Chemical Engineering, East China University of Science and Technology, 130 Meilong Road, Shanghai 200237, China; 2 Shenyang National Laboratory for Materials Science, Institute of Metal Research, Chinese Academy of Sciences, 72 Wenhua Road, Shenyang 110016, China; 3 Department of Chemical Engineering, Norwegian University of Science and Technology, 7491 Trondheim, Norway

**Keywords:** Inorganic Chemistry, Catalysis, Nanomaterials

## Abstract

Development of synergistic heterogeneous catalysts with active sites working cooperatively has been a pursuit of chemists. Herein, we report for the first time the fabrication and manipulation of Pt-WO_3_ dual-active-sites to boost hydrogen generation from ammonia borane. A combination of DFT calculations, structural characterization, and kinetic (isotopic) analysis reveals that Pt and WO_3_ act as the active sites for ammonia borane and H_2_O activation, respectively. A trade-off between the promoting effect of WO_3_ and the negative effect of decreased Pt binding energy contributes to a volcano-shaped activity, and Pt/CNT-5W delivers a 4-fold increased activity of 710.1 mol_H2_·mol_Pt_^−1^·min^−1^. Moreover, WO_3_ is suggested to simultaneously act as the sacrificial site that can divert B-containing by-products away from Pt sites against deactivation, yielding an increase from 24% to 68% of the initial activity after five cycles. The strategy demonstrated here could shed a new light on the design and manipulation of dual-active-site catalysts.

## Highlights

• Mechanism-guided design of Pt-WO_3_ dual active sites boosts H_2_ generation
• A trade-off between WO_3_ loading and Pt B.E. yields a volcano-shaped activity
• WO_3_ acts as the sacrificial site to divert by-products against deactivation
• Multifunctional Pt-MO/C catalysts achieve enhanced activity and durability

## Graphical Abstract

**Figure:**
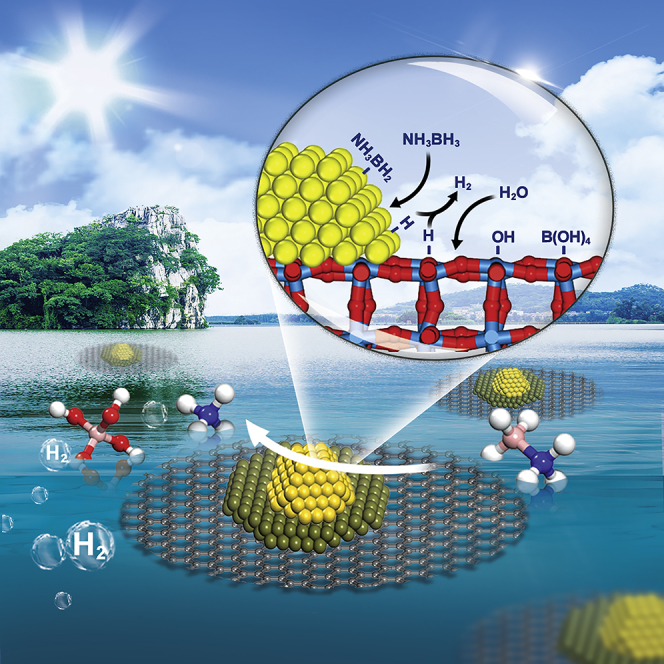

## Introduction

Noble metal catalysts are the workhorses for energy and environment applications, which enable the conversion of feedstock molecules to desired products (Pakhare and Spivey, 2014, Zhou et al., 2010). Nevertheless, their expenses and scarcity limit the viability for large-scale commercialization (Wu et al., 2011, Gong et al., 2012). Continuous efforts have been devoted to engineering the noble metal catalysts by tailoring their sizes, shapes, and compositions to improve the metal utilization efficiency and ultimately the catalytic performance (Aijaz et al., 2012, Zhang et al., 2009, Zecevic et al., 2013). Generally, the supported noble metal catalysts are often chemically and physically complex due to their multi-elemental, porous, and hierarchically structured natures, rendering their rational design and manipulation extremely challenging (Meirer and Weckhuysen, 2018, Yu et al., 2012, Shrestha et al., 2011). Fortunately, with the rapid development of computational chemistry, (micro) kinetics analysis, multiple characterization, isotope experiments, etc., it endows us with great opportunities to explore the reaction mechanism and kinetics with judicious interpretation of their results and then design highly efficient noble metal catalysts (Allian et al., 2011, Aramouni et al., 2018, Chin et al., 2011, Dasgupta et al., 2013, Hmadeh et al., 2014, Norskov et al., 2011, Pan and Bao, 2011, Qiao et al., 2011, Wu et al., 2012, Yamada et al., 2011).

Hydrogen is a well-known ideal energy carrier, and its safe and efficient storage as well as facile release is the key toward a hydrogen economy (Moussa et al., 2013, Turner, 2004, Diwan et al., 2011, Diakov et al., 2007, Demirkan et al., 2019, Sogut et al., 2019, Sen et al., 2018). With high hydrogen content (19.6 wt%), long-term stability, and nontoxicity at room temperature, ammonia borane (NH_3_BH_3_, AB) has been regarded as a promising hydrogen storage material (Yan et al., 2008, Valero-Pedraza et al., 2019, Yao et al., 2016, Li et al., 2017, Metin et al., 2010). Although a fast evolution of hydrogen from ammonia borane hydrolysis has been demonstrated by using noble metal catalyst, especially Pt and Ru, optimizing its activity and durability to minimize its usage is still of paramount importance (Zhang et al., 2017, Zhang et al., 2018, Akbayrak and Ozkar, 2012, Mori et al., 2016, Yan et al., 2017). Some studies have identified the interactions between Pt surface and H atom within ammonia borane and its resultant formation of activated complex species as the prerequisite to generate hydrogen (Chen et al., 2017a, Yang et al., 2011). To this end, it is highly desirable to engineer the properties of metal and substrate through two main approaches. One is alloying with other components to integrate multi-components with different properties (Wang et al., 2014, Zhang et al., 2009, Chen et al., 2017b). The other is tailoring the surface chemistry of catalyst support to obtain the targeted properties of supported metal (Chen et al., 2014a, Chen et al., 2015, Chen et al., 2018, Khalily et al., 2016, Lara et al., 2019).

Notably, rational catalyst design relies on understanding of the mechanism by which catalysts operate. Our recent studies on the kinetics and reaction mechanism of Pt-catalyzed ammonia borane hydrolysis (Chen et al., 2017a) indicate that the Pt catalyst displays a good capacity to dissociate the B–H bond within ammonia borane, but it is intrinsically inactive to dissociate the O–H bond within H_2_O, and the NH_3_BH_2_∗ assisted dissociation of O−H bond within H_2_O∗, i.e., NH_3_BH_2_∗+H_2_O∗→NH_3_BH_2_(OH)∗+H∗ as the rate-determining step (RDS). Moreover, DFT calculations reveal that pure Pt metal surface binds H_2_O too weakly to dissociate H_2_O, whereas some transition metal oxides exhibit great potentials for facile H_2_O dissociation (Ishikawa et al., 2002, Yang et al., 2017). Consequently, it is reasonable to assume that fabricating Pt-metal oxide synergistic catalyst with active sites working cooperatively could pave an effective way for hydrogen generation.

In addition to the hydrogen generation activity, the catalyst durability is another important criterion to evaluate the performance of metal catalysts, often more critical for noble metal catalysts. Our previous studies on highly active carbon-supported Pt catalysts have shown that the catalyst deactivation mainly arises from the agglomeration of Pt nanoparticles and the adsorption of poisonous B-containing by-products on the catalyst surfaces during the reaction (Chen et al., 2014b, Chen et al., 2015). Based on the reaction and deactivation mechanisms, tailoring Pt particle sizes and distributions as well as introducing more oxygen-containing groups and defects onto the carbon support surfaces has been demonstrated to effectively suppress the Pt agglomeration and/or to increase the Pt binding energy for the inhibited poison adsorption (Chen et al., 2014b, Chen et al., 2015, Zhang et al., 2017). Considering that introduction of metal oxides could help stabilize noble metal nanoparticles (Cao et al., 2010) and adsorb anions (Sverjensky and Fukushi, 2006), an attempt would be highly desirable to design Pt-metal oxide multi-functional catalyst by employing the metal oxide to act as anchoring and sacrificial sites against deactivation, thus not only enhancing the hydrogen generation activity mentioned above but also improving the catalyst durability.

Herein, we report a strategy to design and fabricate dual-active-site catalysts to boost hydrogen production from ammonia borane hydrolysis. DFT calculations were first carried out to assist the fabrication of Pt-WO_3_ dual-active-site synergistic catalyst. Along this line, a series of tungsten-incorporated CNT-γW were prepared to immobilize Pt particles with the same loading. Catalytic activity and durability tests were performed to explore the promotion effects of WO_3_ on the catalytic performance. A combination of comprehensive characterizations, kinetic (isotopic) investigations, and DFT calculations was employed to reveal the structure-performance relationship, and a dual-active-site mechanism was proposed to contribute to the simultaneously enhanced activity and durability. This provides a feasible avenue to design and develop dual-active-site catalysts by combining theoretical and experimental studies in this research area.

## Results

### DFT-Assisted Catalyst Design and Fabrication of CNT-γW

**Figure 1:**
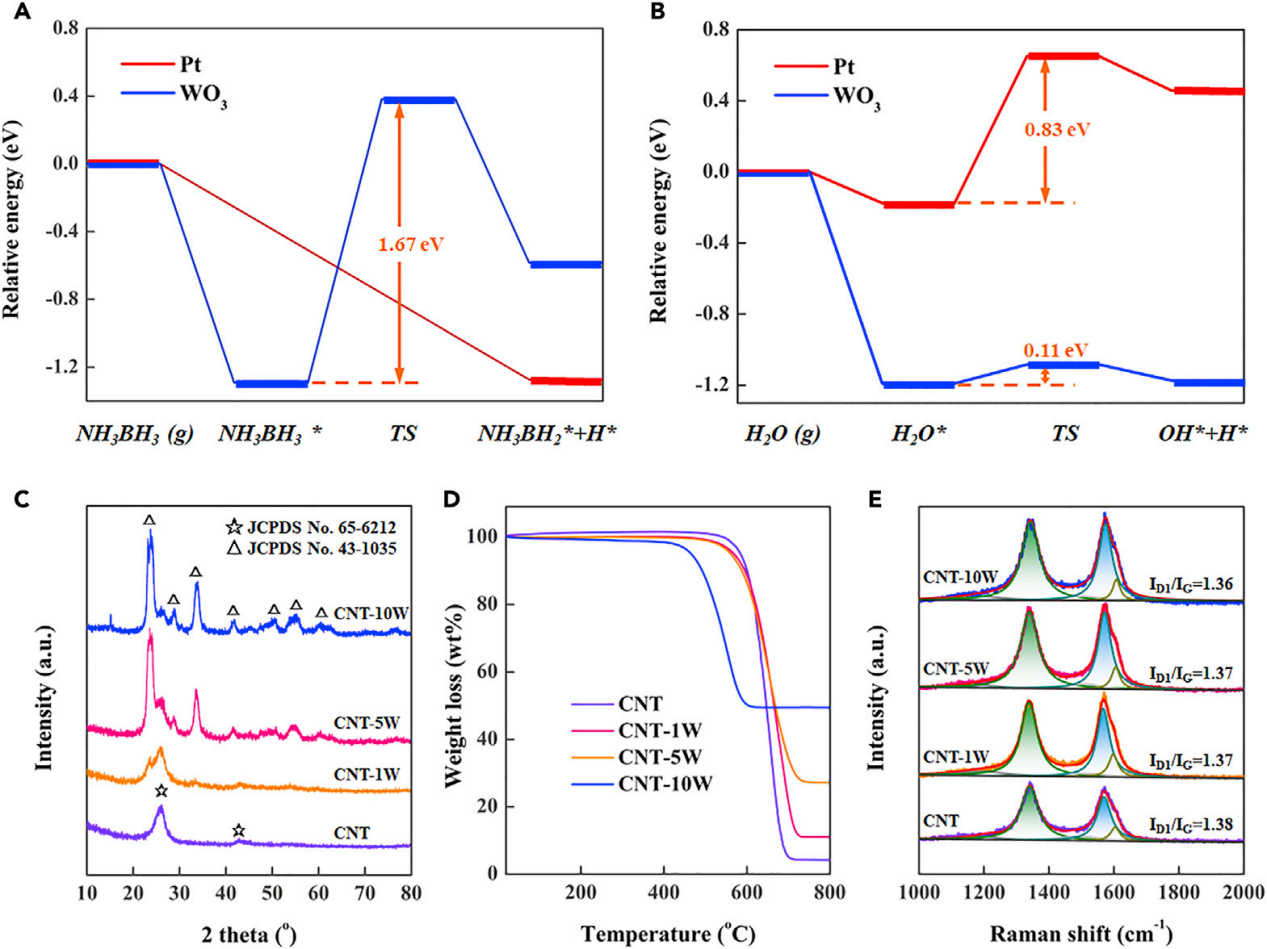
DFT Calculations of Ammonia Borane and H_2_O Activation as well as Structural Characterization of CNT-γW (A) Potential energy diagrams of ammonia borane activation over Pt(111) and WO_3_(100) surfaces. (B) Potential energy diagrams of H_2_O activation over Pt(111) and WO_3_(100) surfaces. (C) XRD patterns of CNT, CNT-1W, CNT-5W, and CNT-10W. (D) TGA profiles of CNT, CNT-1W, CNT-5W, and CNT-10W. (E) Raman spectra of CNT, CNT-1W, CNT-5W, and CNT-10W.

As discussed above, fabricating Pt-metal oxide might be an effective strategy to obtain synergistic catalyst, with dual active sites working cooperatively for the activation of ammonia broane and H_2_O. Exemplified with WO_3_, the adsorption and activation of ammonia broane and H_2_O on the representative WO_3_(100) and Pt(111) surfaces, as the thermodynamically stable and most exposed facets (Hurtado-Aular et al., 2020, Mahata and Pathak, 2017), were comparatively studied by DFT calculations. The optimized most stable adsorption configurations of the involved species on Pt(111) and WO_3_(100) surfaces are listed in Figure S1, and the corresponding potential energy profiles are displayed in Figures 1A and 1B, respectively. It can be obviously observed in Figure 1A that the ammonia borane dissociatively adsorbs on the Pt(111) surface, in comparison with the much higher activation barrier of 1.67 eV over the WO_3_(100) surface. This indicates that the Pt site facilitates the activation of ammonia borane with respect to the WO_3_ site. In contrary, the WO_3_ site (i.e., 0.11 eV) shows much lower activation barrier for H_2_O dissociation than the Pt site (i.e., 0.83 eV) as shown in Figure 1B, in consistent with the larger H-O bond elongation over the WO_3_(100) surface as shown in Figure S1. Therefore, it can be theoretically predicted that fabricating Pt-WO_3_ dual sites for acting as a synergistic catalyst can promote ammonia borane hydrolysis.

Considering that the low specific surface area of WO_3_ is unfavorable for Pt immobilization (Jin et al., 2017), carbon nanotube (CNT) with high external surface area, close ends, and mesoporous structure (Chen et al., 2014b, Chen et al., 2015) was employed as the catalyst support to enhance the specific surface area of WO_3_ by incorporating WO_3_ onto CNT for the following Pt immobilization. Specifically, the tungsten-incorporated supports were prepared by mixing pristine CNT with ammonium tungstate aqueous solutions of different concentrations (i.e., 1, 5 and 10 wt%) at 90°C for 10 h, followed by filtering, washing, drying, and calcination under Ar at 450°C for 2 h. The as-obtained samples were denoted as CNT-γW, in which γ represents the concentration of ammonium tungstate aqueous solution. X-ray diffraction (XRD) spectra in Figure 1C show that, after the incorporation of WO_3_, the CNT-γW samples exhibit some distinct characteristic diffraction peaks of WO_3_ (JCPDS No. 43-1035), in addition to the two characteristic diffraction peaks of graphite (JCPDS No. 65-6212) from the CNT. Notably, these diffraction peaks become intensive and sharp with the concentration of ammonium tungstate solution, indicating the increased amount and size of WO_3_ particles over the CNT-γW.

Thermal gravimetric analysis (TGA) was further carried out to determine the loadings of WO_3_ over the CNT-γW. As shown in Figure 1D, the residue weight of pristine CNT is estimated around 3.0 wt% originating from the metal catalyst of CNT growth, whereas that of CNT-γW samples increases with the concentration of ammonium tungstate aqueous solution. By excluding the weight of CNT growth catalyst, the loadings of WO_3_ over the CNT-1W, CNT-5W, and CNT-10W are determined as around 7.0, 24.1, and 47.2 wt%, respectively. It can also be seen that the onset of carbon support decomposition shifts to low temperature with the loading of WO_3_. This is most likely because the presence of WO_3_ reduces the thermal stability of CNT in air by catalyzing the low-temperature oxidation of CNT, which has been also observed in previous studies (Chiang et al., 2001, Xin and Li, 2011).

**Figure 2:**
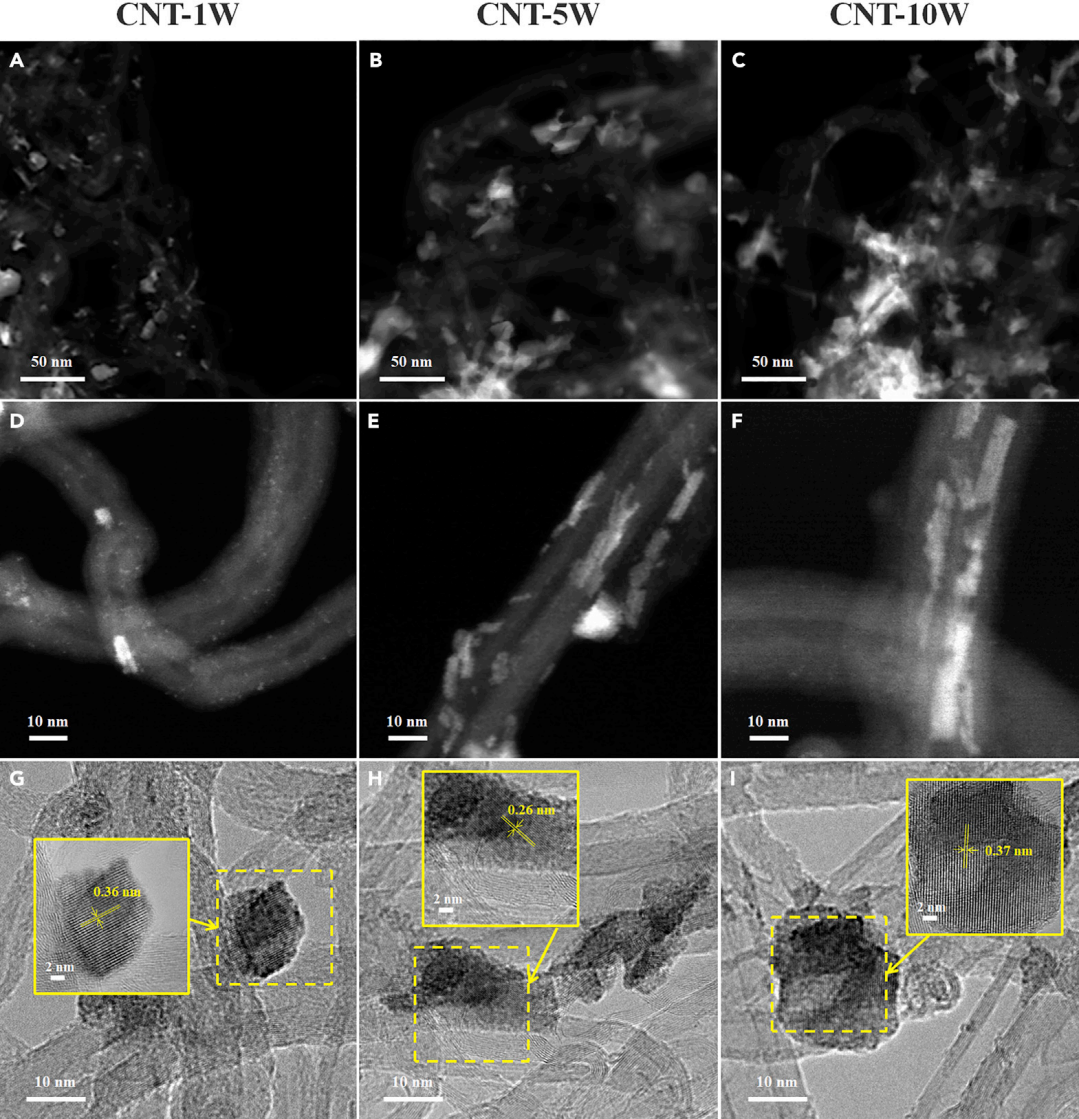
HAADF-STEM and HRTEM Images of CNT-γW (A–C) Low-magnification HAADF-STEM images of (A) CNT-1W, (B) CNT-5W, and (C) CNT-10W. (D–F) High-magnification HAADF-STEM images of (D) CNT-1W, (E) CNT-5W, and (F) CNT-10W. (G–I) HRTEM images of (G) CNT-1W, (H) CNT-5W, and (I) CNT-10W.

Raman measurements were conducted to probe whether the incorporation of WO_3_ affects the surface defects of CNT, for which the intensity ratio of D_1_ band at ∼1340 cm^−1^ to G band at ∼1570 cm^−1^ (I_D1_/I_G_) is used to quantify the surface defects (Sadezky et al., 2005). As shown in Figure 1E, the I_D1_/I_G_ values of CNT, CNT-1W, CNT-5W, and CNT-10W are 1.38, 1.37, 1.37, and 1.36, respectively, indicating neglectable influences of the WO_3_ incorporation on the support surface defects. Low-magnification HAADF-STEM images of CNT-γW in Figures 2A–2C show that the WO_3_ appears as bright patches against the dark carbon matrix background. It can be seen that, for the CNT-1W with low WO_3_ loading, the bright patches distribute homogeneously across the CNT surface in small sizes. With the increase of WO_3_ loading, the density and size of these patches increase to high levels. In the high-magnification image of Figure 2D, some tiny WO_3_ nanoparticles appear as bright dots on the CNT-1W, which could be due to the low tungsten loading as well as the strong interaction between WO_3_ and CNT. In comparison, Figures 2E and 2F reveal that the CNT-5W and CNT-10W mainly consist of large strip-shaped particles along the CNT wall. Moreover, the HRTEM images in Figures 2G–2I exhibit continuous ordered lattice fringes, and the lattice spacings of ∼0.36 and ∼0.26 nm correspond to the (200) and (202) facets of WO_3_, respectively. These results are in good agreement with XRD results that the tungsten oxide species are mainly in the form of WO_3_, and the amount and size of WO_3_ particles sharply increases with the WO_3_ loading.

### Synthesis and Structural Characterization of Pt-WO_3_ Dual Sites

**Figure 3:**
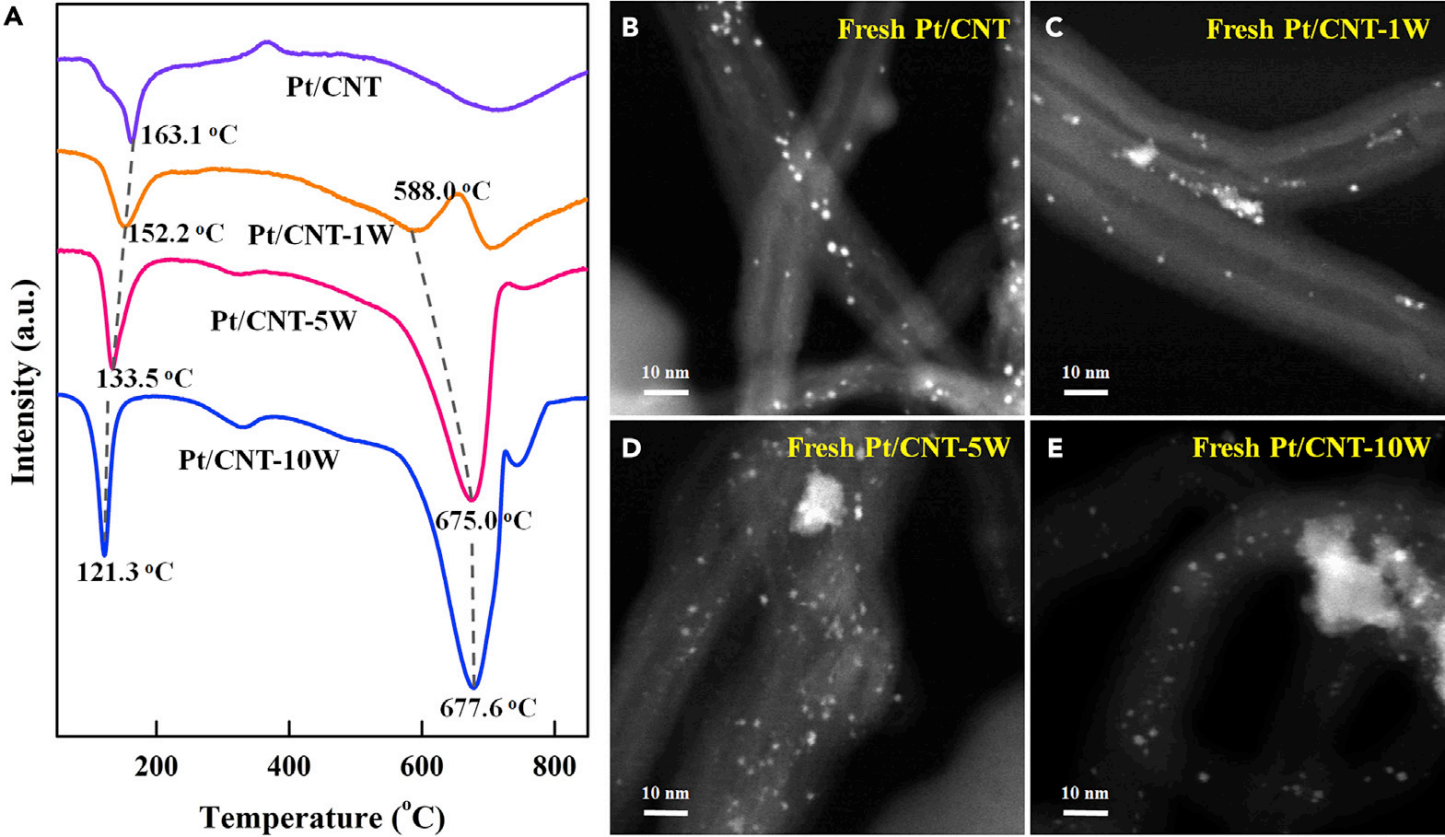
H_2_-TPR Profiles and HAADF-STEM Images of Catalysts (A) H_2_-TPR profiles of Pt/CNT, Pt/CNT-1W, Pt/CNT-5W, and Pt/CNT-10W. (B–E) Typical HAADF-STEM images of the fresh (B) Pt/CNT, (C) Pt/CNT-1W, (D) Pt/CNT-5W, and (E) Pt/CNT-10W catalysts.

The above fabricated-tungsten-incorporated CNT-γW samples as well as the pristine CNT as a reference were impregnated with H_2_PtCl_6_ solutions to prepare the catalysts, with an aim to construct Pt-WO_3_ dual active sites. H_2_ temperature-programmed reduction (H_2_-TPR) measurement was first conducted to explore the interactions among Pt, WO_3_, and CNT. As shown in Figure 3A, for the pristine-CNT-supported Pt catalyst, two hydrogen consumption peaks could be observed at 163.1 and 709.3°C, which could be due to the reduction of platinum species and methanation of CNT support, respectively. However, for the Pt/CNT-γW catalysts, another new hydrogen consumption peak between the above two peaks is observed and ascribed to the reduction of WO_3_. Notably, the increasing WO_3_ loading leads to the upshift of WO_3_ reduction peak, which eventually overlaps with the methanation peak, possibly due to the formation of larger WO_3_ particles in Figures 1C and 2. Accordingly, the Pt species reduction peak shifts to low temperature with increasing WO_3_ loading, resulting from the interaction of Pt with WO_3_.

HAADF-STEM was employed to characterize the Pt particle size and distribution of these catalysts. Figure 3B reveals that the Pt particles on pristine CNT show a relatively homogeneous distribution, and the average Pt particle size based on the measurements of more than 200 random particles is determined to be 1.4 nm. In comparison, the Pt/CNT-1W in Figure 3C exhibits the coexistence of a few small patches, which could be WO_3_ clusters, with some small spots, which could be Pt and WO_3_ nanoparticles. Considering that the contrast variations in HAADF-STEM characterization are proportional to the square of the atomic number (Van Aert et al., 2011), the brighter and less bright spots could be ascribed to Pt and WO_3_ nanoparticles, respectively. Therefore, it can be seen that a majority of Pt nanoparticles remain on the graphitic wall of CNT, whose sizes are similar to those of Pt/CNT, and a few bright Pt spots are observed on the WO_3_ clusters.

**Figure 4:**
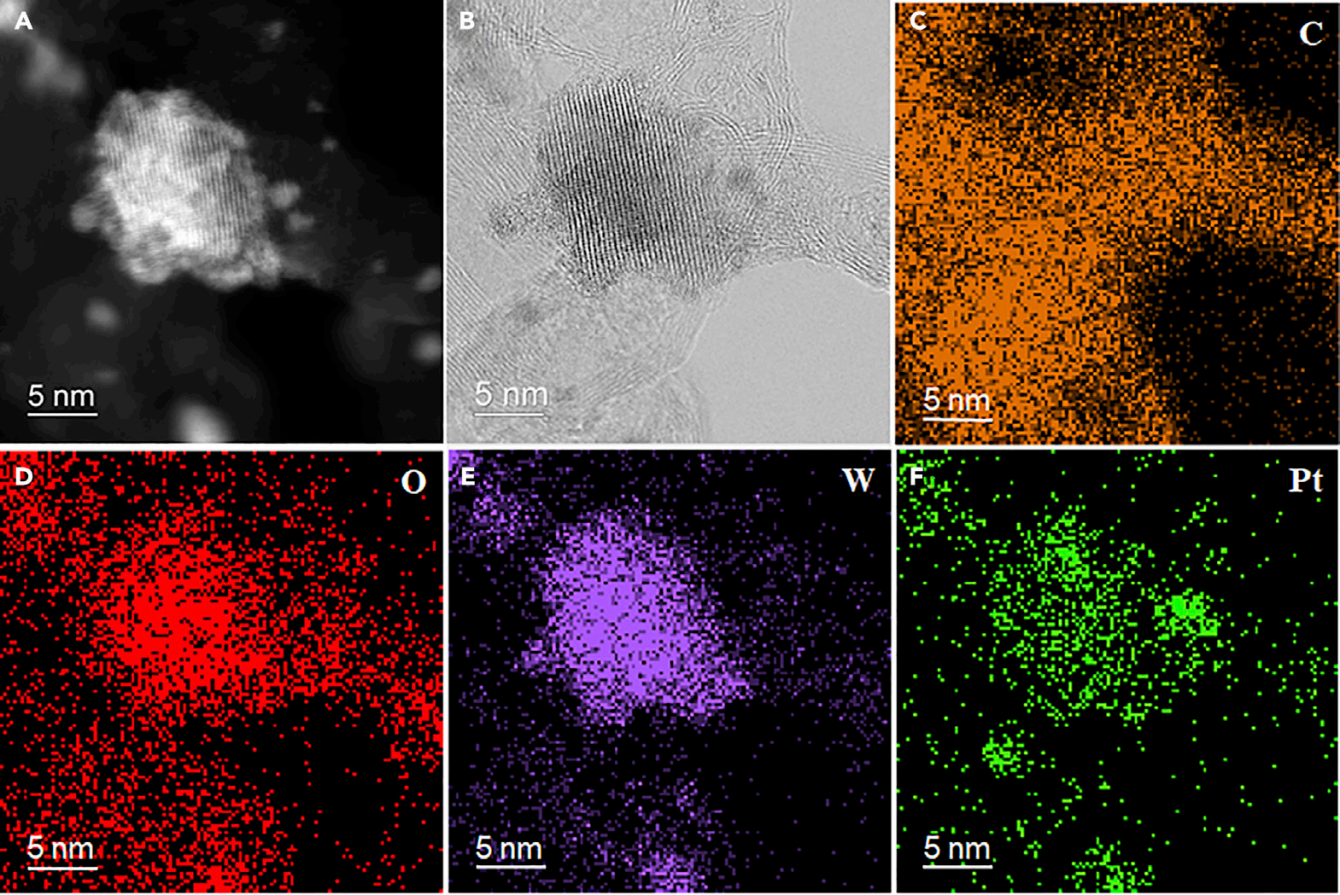
Atomic Distribution Characterization of Pt/CNT-5W Catalyst (A) Typical HAADF-STEM image of Pt/CNT-5W. (B) Typical HRTEM image of Pt/CNT-5W. (C–F) The corresponding EDS mappings of (C) C, (D) O, (E) W, and (F) Pt elements.

As shown in Figures 3D and 3E, with the increase of WO_3_ loading, the density of bright spots becomes intensive for Pt/CNT-5W and Pt/CNT-10W, making it difficult to distinguish Pt from WO_3_ particles. Hence, energy dispersive spectroscopy (EDS) mapping of the selected areas in Figures 4A and 4B was further conducted to analyze the elemental distributions, and the results are shown in Figures 4C–4F. The EDS mapping of oxygen and tungsten in Figures 4D and 4E reveals that the Pt/CNT-5W mainly consists of large WO_3_ patches as well as a few dispersed particles, consistent with the HAADF-STEM results. Interestingly, as shown in Figure 4F, the EDS mapping of Pt suggests that the Pt particles mainly concentrate on WO_3_ patches instead of on CNT. Considering that the electron microscopic characterization could only reflect the local information of the sample, another two areas as depicted in Figures S2 and S3 were chosen by the same method to characterize this sample, so as to reduce the errors induced by the selected areas. Obviously, a majority of Pt particles still interact with WO_3_ patches rather than with CNT, and the corresponding Pt particle sizes still remain in the range of 1–2 nm, which is comparable to that for the pristine CNT. Hence, all the above results indicate that the Pt particles prefer to interact with the WO_3_ patches, which is consistent with the decreased reduction temperature as shown in Figure 3A.

**Figure 5:**
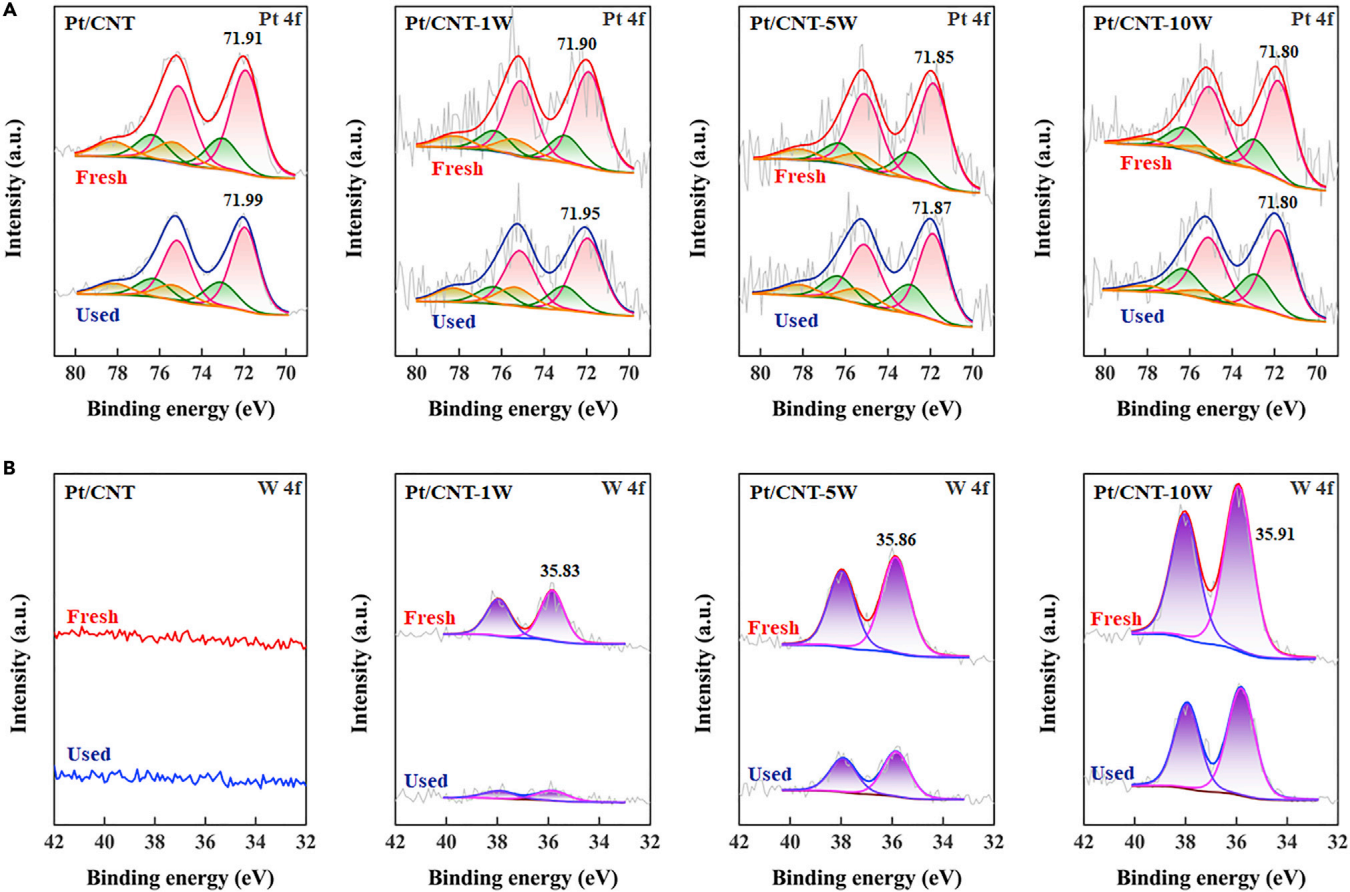
XPS Characterization of the Fresh and Used Catalysts (A) Pt 4f spectra of the fresh and used catalysts. (B) W 4f spectra of the fresh and used catalysts.

XPS was employed to investigate the electronic properties of these catalysts, and the results are shown in Figure 5. The Pt 4f region of XPS spectra in Figure 5A shows two intense peaks corresponding to Pt 4f_7/2_ and Pt 4f_5/2_, which can be deconvoluted into three pairs of doublets, i.e., Pt^0^, Pt^2+^, and Pt^4+^. Table S1 summarizes the binding energy (B.E.) as well as the corresponding percentage of Pt species. It can be seen that all the catalysts exhibit similar percentages of Pt^0^, which has been identified as the main active species for this reaction (Chandra and Xu, 2006). Interestingly, it is found that the Pt B.E. shifts to lower value with the WO_3_ loading. Correspondingly, the deconvolution of W 4f region in Figure 5B shows an opposite trend of W B.E. Considering the similar Pt particle sizes for these catalysts, the observed Pt B.E. downshift and W B.E. upshift are mainly ascribed to electron transfer between Pt and WO_3_. Specifically, WO_3_ could act as an electron donor and transfer electrons to Pt, giving rise to the electron-rich Pt particles with lower Pt B.E. Hence, with the increase of Pt-WO_3_ interactions, more and more electrons are transferred to Pt particles, resulting in the continuous decrease of Pt B.E. and increase of W B.E. for the Pt/CNT-γW catalysts.

### Kinetic (Isotopic) and Durability Analyses

**Figure 6:**
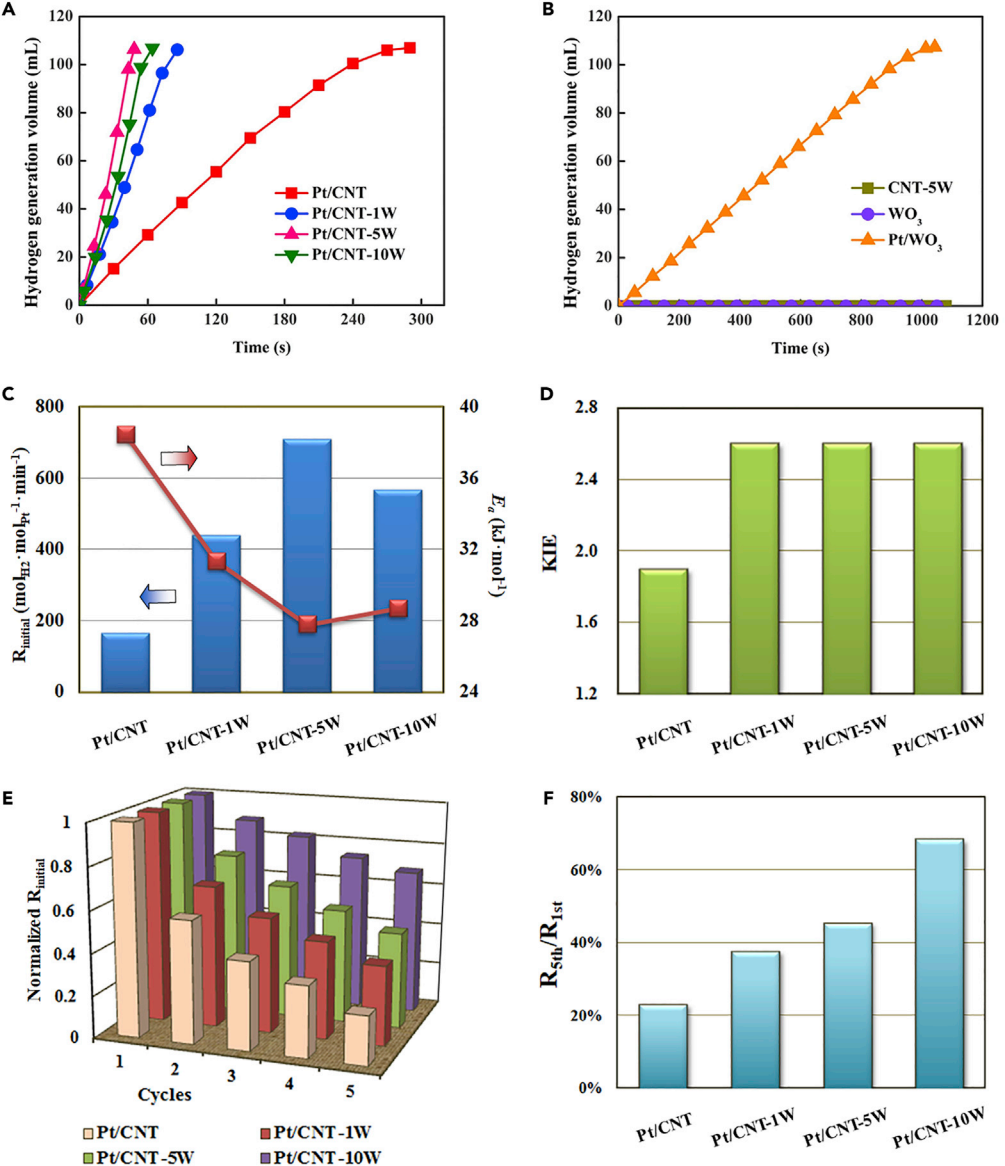
Catalytic Activity and Durability as well as Kinetics Analysis (A) Hydrogen generation as a function of time for Pt/CNT, Pt/CNT-1W, Pt/CNT-5W, and Pt/CNT-10W at 30°C. (B) Hydrogen generation as a function of time for CNT-5W, WO_3_, and Pt/WO_3_ at 30°C. (C) R_initial_ and *E*_*a*_ of Pt/CNT, Pt/CNT-1W, Pt/CNT-5W, Pt/CNT-10W, and CNT-5W. (D) Kinetic isotope effect (KIE) values of Pt/CNT, Pt/CNT-1W, Pt/CNT-5W, and Pt/CNT-10W. (E) The relative activities over cycles of Pt/CNT, Pt/CNT-1W, Pt/CNT-5W, and Pt/CNT-10W. (F) The ratio of the activity in the fifth cycle to that in the first run, R_5th_/R_1st_, for Pt/CNT, Pt/CNT-1W, Pt/CNT-5W, and Pt/CNT-10W.

Catalytic behaviors of these Pt-WO_3_ dual sites catalysts together with the reference Pt/CNT catalyst and CNT-5W were explored for ammonia borane hydrolysis, and the results are shown in Figures 6A and 6B. Obviously, the volume of hydrogen generation is proportional to the reaction time at the initial reaction stage (i.e., AB conversion lower than 45 ± 5%), suggesting pseudo-zero order kinetics for the reaction. As a result, the corresponding initial reaction rate (R_initial_) can be calculated based on the slope of linear part for each plot in Figure 6A. As shown in Figure 6C, the R_initial_ values of Pt/CNT, Pt/CNT-1W, Pt/CNT-5W, and Pt/CNT-10W catalysts are determined as 165.2, 439.2, 710.1, and 557.5 mol_H2_·mol_Pt_^−1^·min^−1^, respectively. By combining previous results that the high Pt B.E. is favorable for ammonia borane hydrolysis (Chen et al., 2014a, Chen et al., 2015), the Pt/CNT-γW catalysts with lower Pt B.E. than the Pt/CNT catalyst should give a lower hydrogen generation rate from the perspective of electronics, which is contradictory to the observation in Figure 6A. This strongly indicates that the fabrication of Pt-WO_3_ dual sites is favorable for ammonia borane hydrolysis, verifying the above theoretical prediction of Pt-WO_3_ acting as the dual active sites for this reaction.

To gain more insights into the reaction over such Pt-WO_3_ dual active sites, kinetics analyses of Pt/CNT-γW against Pt/CNT were performed, and the results are shown in Figures S4 and 6C. Clearly, the Pt/CNT-γW catalysts show lower activation energy (*E*_*a*_) than the Pt/CNT catalyst, and the trend of *E*_*a*_ is consistent with that of R_initial_ mentioned above. This suggests that the fabrication of Pt-WO_3_ dual active sites contributes to the enhanced kinetics. Furthermore, kinetic isotope experiments by replacing H_2_O with D_2_O as the reactant were conducted to probe the kinetic isotope effects and further the reaction mechanism (Chen et al., 2000), and the results are shown in Figures S5 and 6D. All the Pt/CNT-γW catalysts exhibit the similar k_H_/k_D_ values (∼2.6), which are higher than that of the Pt/CNT catalyst (1.9). The change in the k_H_/k_D_ is most likely ascribed to the change in the active sites, i.e., the single Pt site for the reaction over the Pt/CNT catalyst, but the dual Pt and WO_3_ sites for the reaction over the Pt/CNT-γW catalysts.

In addition, it was further explored whether the fabricated Pt/CNT-γW catalysts could also improve the catalytic durability. Herein, the catalyst durability was investigated by adding an equivalent ammonia borane solution after the completion of last cycle. As shown in Figure S6, all the catalysts show decreased catalytic activity as a function of catalytic cycle with different extents. To make a clear comparison, the reaction rate in each cycle was normalized to that of the first cycle. It can be seen in Figures 6E and 6F that the incorporation of WO_3_ remarkably promotes the catalytic durability of Pt/CNT, which increases with the WO_3_ loading. The pH values of the reaction solutions were firstly measured in the range of 9.1–9.8 for all the catalysts during the reaction. This could be ascribed to the acid-base equilibrium of B(OH)_4_^-^⇋B(OH)_3_+OH^−^ for the B-containing byproducts in the reaction solution (Chen et al., 2017a), considering that the p*K*_*a*_ value of acid-base couple of B(OH)_3_–B(OH)_4_^-^ is 9.2 (Sanyal et al., 2011). Thus the similar pH values for the catalysts could help exclude their influences as the main reason for the significantly improved durability. To gain more insights, multiple characterizations were carried out to compare the catalyst properties before and after the durability test. HAADF-STEM measurements in Figures S7 and 3 indicate that some Pt agglomerations are observed after the durability test over Pt/CNT and Pt/CNT-1W, whereas no obvious agglomerations for Pt/CNT-5W and Pt/CNT-10W. This is most likely due to the strong interaction of Pt with WO_3_ to suppress Pt agglomeration, being one factor for the higher catalyst durability of the Pt/CNT-5W and Pt/CNT-10W catalysts.

As mentioned above, the adsorption of B-containing by-products on the Pt surfaces is another main factor leading to the catalyst deactivation (Chen et al., 2014b). The XPS spectra in Figure 5A and their deconvolution results in Table S1 show that the used Pt catalysts exhibit positive shifts in Pt B.E. compared with the fresh ones, mainly ascribed to the electron-deficient nature of the adsorbed B-containing by-products (Chen et al., 2018). Considering that the change in XPS signal intensity is proportional to the concentration of a given element per unit overlayer (Matrab et al., 2007, Precht et al., 2016), it has been previously used in our work (Chen et al., 2015) to compare the amount of species on the Pt surface. As shown in Figure 5, the used catalysts show slightly decreased XPS signal intensities for the Pt spectra but remarkably decreased ones for the W spectra in comparison with the fresh catalysts. This strongly suggests a preferential adsorption of B-containing by-products on the WO_3_ surface during the reaction. In other words, WO_3_ acts as the sacrificial site to preferentially adsorb B-containing by-products, which can suppress the adsorption of B-containing by-products on the Pt surfaces. Further combining the Pt/CNT-10W catalyst with the highest WO_3_ loading, it would provide a rational interpretation for the Pt/CNT-10W catalyst with the highest durability, i.e., retaining 68% of its initial activity at the fifth cycle (R_5th_/R_1st_) in comparison with 24% for the Pt/CNT catalyst, which mainly arises from the most sacrificial sites of WO_3_ for the adsorption of B-containing by-products.

### Mechanism of Pt-WO_3_ Dual Sites for Enhanced Activity and Durability

As described above, DFT calculations show that the activation of ammonia borane reactant proceeds by its dissociative adsorption on the Pt site, and the WO_3_ site exhibits much lower activation barrier for H_2_O dissociation than on the Pt site. On a single Pt active site, ammonia borane hydrolysis over the Pt/CNT catalyst has been demonstrated to proceed by the NH_3_BH_2_∗-assisted cleavage of O-H bond within H_2_O (Chen et al., 2017a). Interestingly, on the Pt-WO_3_ dual active sites, the kinetics (isotope) analysis shows significantly decreased activation energy of the reaction, but an increase in k_H_/k_D_ ratio from 1.9 to 2.6, which contribute to the enhanced kinetics for the reaction over the Pt/CNT-γW catalysts.

The strategy demonstrated above has been successfully developed to fabricate high specific surface area of WO_3_ by incorporating it onto the CNT with larger specific surface area, close ends, and mesoporous structure, which is a promising candidate to immobilize Pt for favorably constructing Pt-WO_3_ dual active sites and enhancing hydrogen generation activity. If directly using commercial WO_3_ (usually having relatively low specific surface area) as the catalyst or catalyst support, the catalyst testing results in Figure 6B show that the WO_3_ is almost inactive for the reaction, and the Pt/WO_3_ catalyst gives much lower hydrogen generation rate than the Pt/CNT-γW catalysts, mainly due to the larger Pt particle size and undesirable Pt B.E in Figure S8. Therefore, the fabrication of Pt-WO_3_ immobilized on the CNT is suggested as an effective way to obtain more Pt-WO_3_ dual active sites for the promoted reaction.

In our previous studies (Chen et al., 2014a, Chen et al., 2017b), it has been found that the electron-deficient Pt particles with the higher Pt B.E. facilitates the adsorption and activation of the reactants toward fast hydrogen evolution. In the present study, the incorporation of WO_3_ not only leads to the Pt/CNT-γW catalysts with lower Pt B.E. than the Pt/CNT catalyst, unfavorable for the reaction, but also fabricates highly active Pt-WO_3_ dual active sites, favorable for the reaction. Such trade-off between the promotion effects of WO_3_ and the negative electronic effects of Pt B.E. can pave an explanation for the observed volcano-shaped activity in Figure 6C.

In addition, the incorporation of WO_3_ also demonstrates remarkable enhancements in the durability. This is attributed to the preferential adsorption of B-containing by-products over the WO_3_ sites. As the reaction proceeds, the produced B-containing by-products would transfer and accumulate on the WO_3_ site instead of the Pt site. Considering that WO_3_ is in large excess, the accumulation of B-containing by-products has much less influence on the activity. Therefore, the WO_3_ site is also suggested as the sacrificial site, diverting B-containing by-products away from the Pt site during the reaction. With the increase of WO_3_ loading, more and more Pt sites in contact with WO_3_ sites are sterically protected from deactivation.

**Scheme 1:**
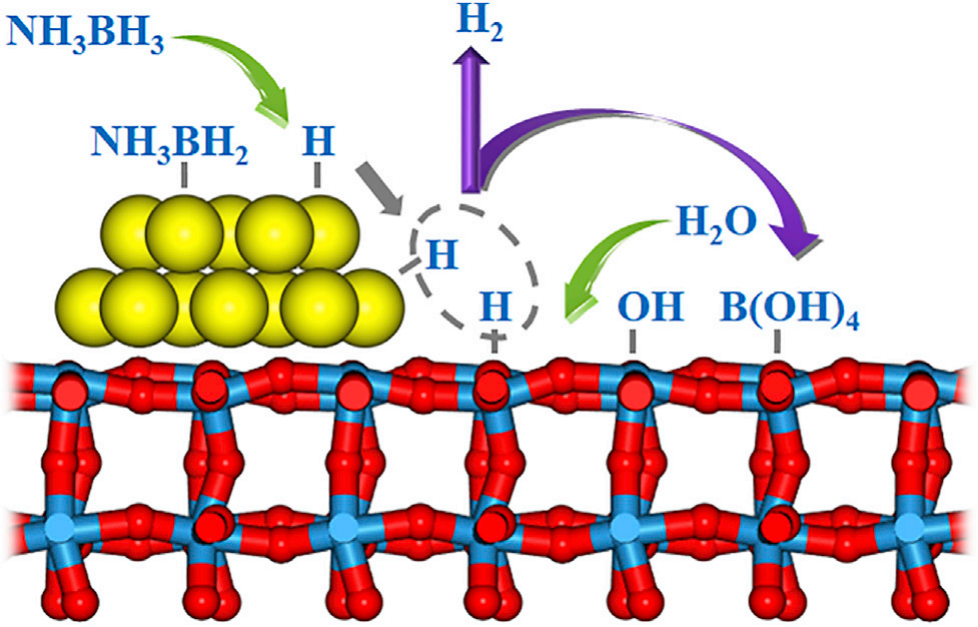
A Proposed Mechanism for Ammonia Borane Hydrolysis over Pt-WO_3_ Dual Metal Sites

Based on the discussion of the mechanism of Pt-WO_3_ dual sites for the enhanced activity and durability, Scheme 1 is proposed to mainly illustrate ammonia borane hydrolysis over Pt-WO_3_ dual active sites, in which the Pt site and WO_3_ site contribute to the activation of ammonia borane and H_2_O, respectively. On the other hand, the WO_3_ site also acts as the sacrificial site to preferentially adsorb B-containing by-products, which can suppress the adsorption of B-containing by-products on the Pt surfaces. Such cooperativity between Pt and WO_3_ sites creates a unique synergy and demonstrates robust hydrogen generation from ammonia borane hydrolysis.

In addition, Table 1 gives a comparison of the activity and durability of Pt/CNT-W catalysts developed in this study with various monometallic Pt-based catalysts in the literature. Obviously, the Pt/CNT-W catalysts show a superior hydrogen generation activity in addition to relatively high durability. Our previous studies have identified Pt(111) as the dominant active sites for the reaction, whose number reaches the optimal value at the mean Pt particle size of ∼1.8 nm (Chen et al., 2014b); and the high Pt B.E. is favorable for both the hydrogen generation activity and durability (Chen et al., 2014a). Thus, the fabricated Pt-WO_3_ dual sites enhance not only the hydrogen generation activity and kinetics due to the Pt-WO_3_ dual active sites but also the durability due to the sacrificial site of WO_3_ for the preferential adsorption of B-containing byproducts during the reaction. Moreover, it can be seen in Table 1 that the employment of carbon support plays a crucial role in Pt electronic properties to remarkably increase the catalytic activity, and the dual sites of Pt-metal oxides (MO, e.g., CeO_2_, Fe_3_O_4_ and TiO_2_) endow the catalysts with a significantly enhanced durability. All of these discussions could shed a new light on the rational design and manipulation of highly active and durable Pt-based catalysts for the reaction, e.g., fabricating Pt-MO/C multifunctional catalysts with appropriate transition metal and carbon toward significantly enhanced hydrogen generation activity and durability.

**Table 1:** A Comparison of the Activity and Durability over Monometallic Pt-Based Catalysts for Ammonia Borane Hydrolysis

Catalyst	d_Pt_ (nm)	Pt B.E. (eV)	R (mol_H2_·mol_M_^−1^·min^−1^)	*E*_*a*_ (kJ·mol^−1^)	Durability	Ref.
Commercial Pt/C	2.5	–	83.3^a^^,^^b^	–	–	Chandra and Xu, 2007
Pt/C	1.9	–	111^a^^,^^b^	–	–	Chandra and Xu, 2007
Pt(8%)/CCF-500	3.4	71.80	35^c^	39.2	47% (r_5th_/r_1st_)^a^	Yuan et al., 2017
(Zn’_6_)Pt/RGO	1.2	∼71.5	284^b^	–	49% (r_5th_/r_1st_)^a^	Chen et al., 2017c
SiO_2_@Pt@NGO	1.9	71.12	324.6^c^	–	39% (r_6th_/r_1st_)	Ye et al., 2017
Pt20/CNT	1.9	71.5	416.5^b^	48.3	40% (r_4th_/r_1st_)	Zhang et al., 2017
Pt/CNT-G	2.8	–	135^b^	35.3	–	Uzundurukan and Devrim, 2019
Pt/CNT-P	1.4	71.9	141.7^c^	–	24% (r_5th_/r_1st_)	Chen et al., 2015
Pt/CNT-O	1.6	71.7	54.4^c^	–	~43% (r_5th_/r_1st_)	Chen et al., 2015
Pt/CNT-D	1.3	72.0	416.0^c^	–	~62% (r_5th_/r_1st_)	Chen et al., 2015
Pt/CNT-5W	~1.4	71.85	710^c^	27.8	45% (r_5th_/r_1st_)	This work
Pt/CNT-10W	~1.4	71.80	558^c^	28.7	68% (r_5th_/r_1st_)	This work
Pt-CeO_2_/rGO	2.8	71.26	93.8^b^	64.7	92% (r_10th_/r_1st_)	Yao et al., 2016
Pt/SiO_2_	5.1	–	55^a^	–	–	Chandra and Xu, 2007
Pt@MIL-101	1.8	–	414^b^	–	–	Aijaz et al., 2012
Fe_3_O_4_@SiO_2_@Pt@mSiO_2_	–	–	5.5^d^	35.4	62% (r_5th_/r_1st_)^a^	Xu et al., 2017
Pt-CeO_2_	5.0	–	133^b^	–	66% (r_5th_/r_1st_)	Wang et al., 2012
SEA-Pt/HNTs	1.5	–	321^b^	49.2	71% (r_10th_/r_1st_)	Yin et al., 2019
Pt25@TiO_2_	2.4	71.02	311^b^	–	75% (r_3rd_/r_1st_)	Khalily et al., 2016

a Estimated from the slope of the fitting line.

b Measured under 25°C.

c Measured under 30°C.

d Measured under 35°C.

## Discussion

In summary, we report for the first time the fabrication and manipulation of multifunctional Pt-WO_3_/CNT catalysts with simultaneously enhanced hydrogen generation activity and durability. A combination of DFT calculations and multiple characterizations with kinetic (isotopic) analysis demonstrates that the Pt-WO_3_ acts as the dual active sites for the activation of ammonia borane and H_2_O. By incorporating the WO_3_ onto CNT to immobilize Pt particles, the resultant Pt/CNT-γW catalysts give rise to more Pt-WO_3_ dual active sites with the desirable Pt B.E. than the commercial WO_3_-supported Pt catalyst. By increasing WO_3_ loading, a trade-off between the promotion effect of WO_3_ and the negative electronic effect of decreased Pt binding energy contributes the volcano-shaped activity, in which the Pt/CNT-5W delivers the highest catalytic activity of 710.1 mol_H2_·mol_Pt_^−1^·min^−1^, more than four folds higher than that of pristine Pt/CNT. On the other hand, the WO_3_ site acts as the sacrificial site and diverts B-containing by-products away from the Pt site, thus inhibiting the catalyst deactivation during the reaction and yielding a significant increase from 24% to 68% of the initial catalytic activity after five cycles. This report not only shows a high potential to achieve robust hydrogen generation from ammonia borane hydrolysis but also guides the rational design and manipulation of dual-site catalysts with the multifunctional properties by combining theoretical and experimental studies.

### Limitations of the Study

Currently, it is very difficult for this research to precisely characterize the microstructures of the fabricated highly active and durable Pt/CNT-γW catalysts at an atomic level. This would be facilitated by advanced in-situ/ex-situ catalyst characterization, such as Cs-corrected TEM and XAS. Then, more desirable theoretical models are needed for in-depth understanding of the reaction mechanism and kinetics to reveal the underlying dual-active-site mechanism.

## Methods

All methods can be found in the accompanying Transparent Methods supplemental file.

## Supplemental Information

Document S1. Transparent Methods, Figures S1–S8, and Table S1
