# Supplementary material for: Synergistic Pt-WO3 Dual Active Sites to Boost Hydrogen Production from Ammonia Borane
Source: iScience. 2020 Feb 19;23(3):100922. doi: 10.1016/j.isci.2020.100922 (PMC7052519; doi:10.1016/j.isci.2020.100922)
Supplement: Document S1. Transparent Methods, Figures S1–S8, and Table S1 [file mmc1.pdf]

iScience, Volume *23*

Supplemental Information

Synergistic Pt-WO_3 Dual Active Sites to Boost Hydrogen Production from Ammonia Borane

Wenyao Chen, Wenzhao Fu, Gang Qian, Bingsen Zhang, De Chen, Xuezhi Duan, and Xinggui Zhou

Supplemental Table

Table S1. The binding energy (B.E.) as well as corresponding percentage of Pt species determined by XPS for the fresh and used catalysts, related to Figure 5.

Catalyst		Pt^n $4f_{7/2}$ B.E. (eV)			Pt^n percentage		
		Pt^0	Pt^{2+}	Pt^{4+}	Pt^0	Pt^{2+}	Pt^{4+}
Pt/CNT	Fresh	71.91	73.00	75.30	64.4%	20.7%	14.9%
	Used	71.99	73.03	75.31	63.4%	21.6%	15.0%
Pt/CNT-1W	Fresh	71.90	73.00	75.30	67.7%	19.2%	13.1%
	Used	71.95	73.05	75.35	61.9%	20.2%	17.9%
Pt/CNT-5W	Fresh	71.85	72.95	75.26	69.7%	18.6%	11.7%
	Used	71.87	72.95	75.30	62.6%	24.0%	13.4%
Pt/CNT-10W	Fresh	71.80	72.90	75.23	70.3%	22.4%	7.3%
	Used	71.80	72.90	75.28	64.9%	27.7%	7.4%

Supplemental Figures

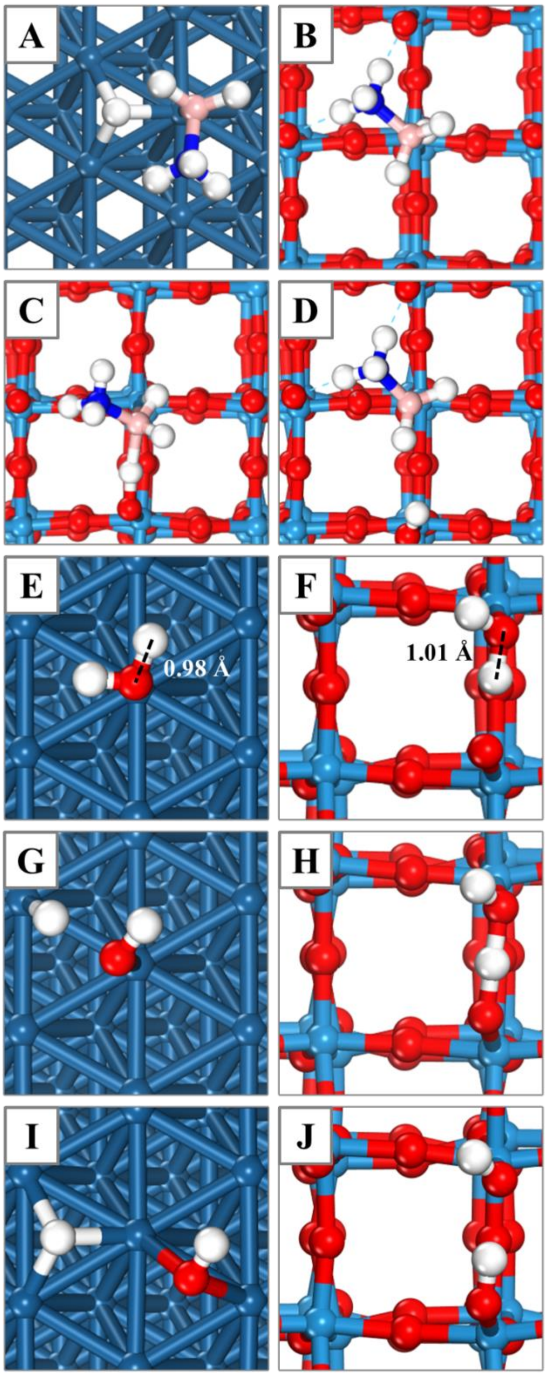

Figure S1. The optimized most stable configurations, related to Figure 1. (A) The final (NH_3BH_2*+H*) state over Pt(111) surface. (B) The initial (NH_3BH_3*), (C) transition (TS) and (D) final (NH_3BH_2*+H*) states over WO_3(100) surface. The (E, F) initial (H_2O*), (G, H) transition (TS) and (I, J) final (HO*+H*) states over Pt(111) and WO_3(100) surface, respectively. Blue, white, red, pink, dark blue and light blue spheres are Pt, H, O, N, B and W atoms, respectively.

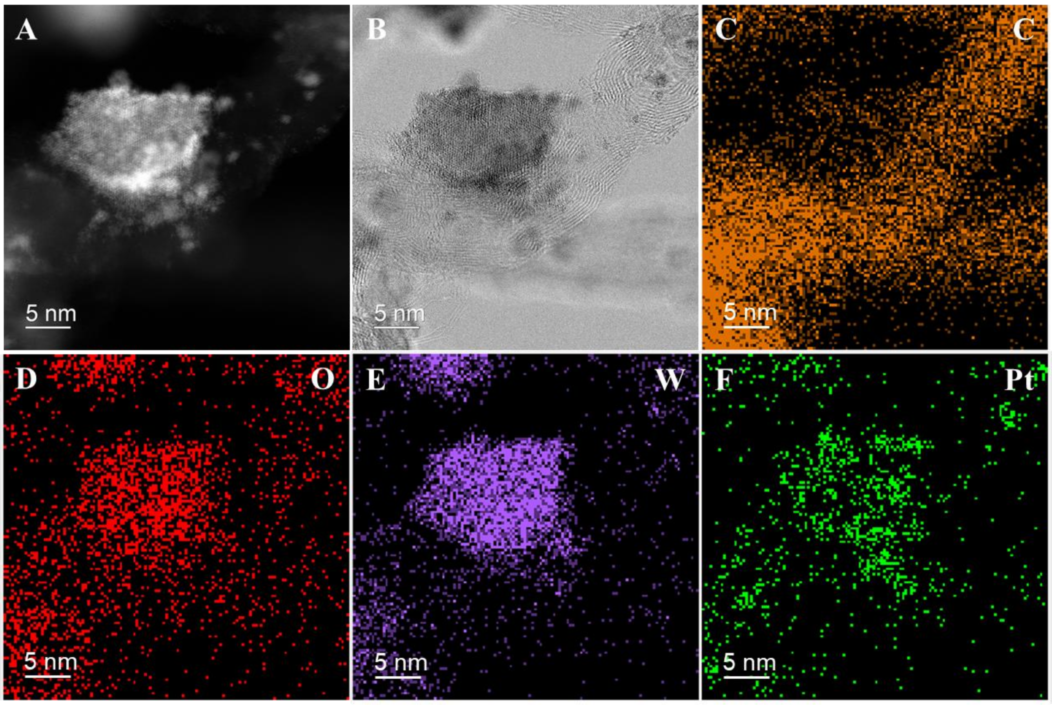

Figure S2. Atomic distribution characterization of Pt/CNT-5W, related to Figure 4. (A) Typical HAADF-STEM image of Pt/CNT-5W. (B) Typical HRTEM image of Pt/CNT-5W. (C-F) The corresponding EDS mappings of C, O, W and Pt elements.

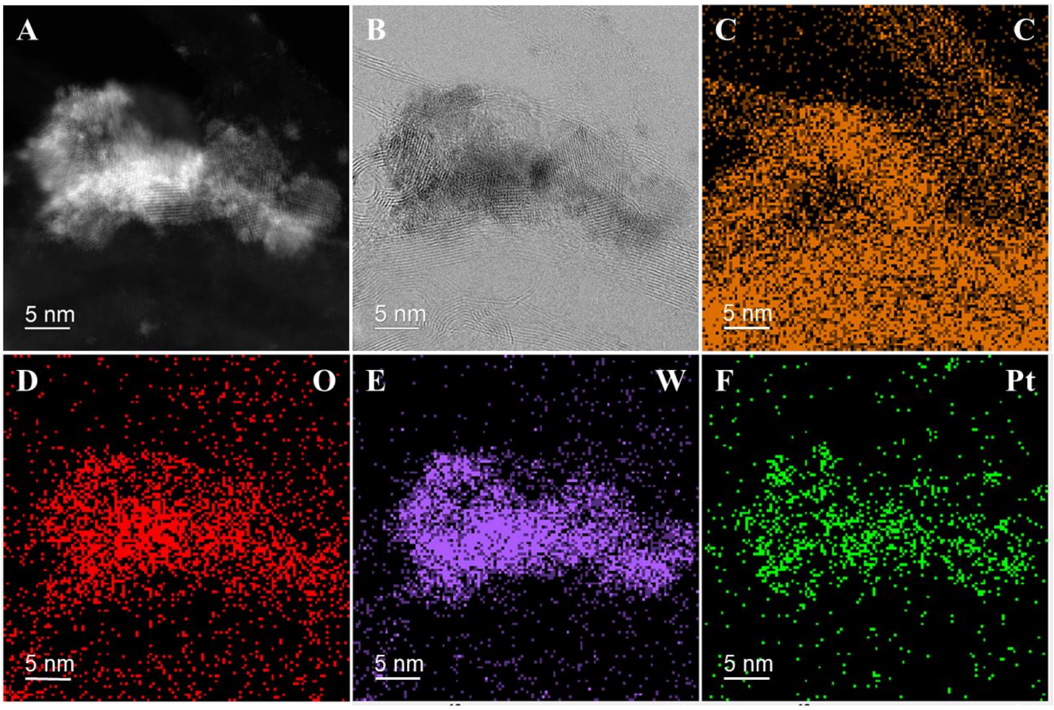

Figure S3. Atomic distribution characterization of Pt/CNT-5W, related to Figure 4. (A) Typical HAADF-STEM image of Pt/CNT-5W. (B) Typical HRTEM image of Pt/CNT-5W. (C-F) The corresponding EDS mappings of C, O, W and Pt elements.

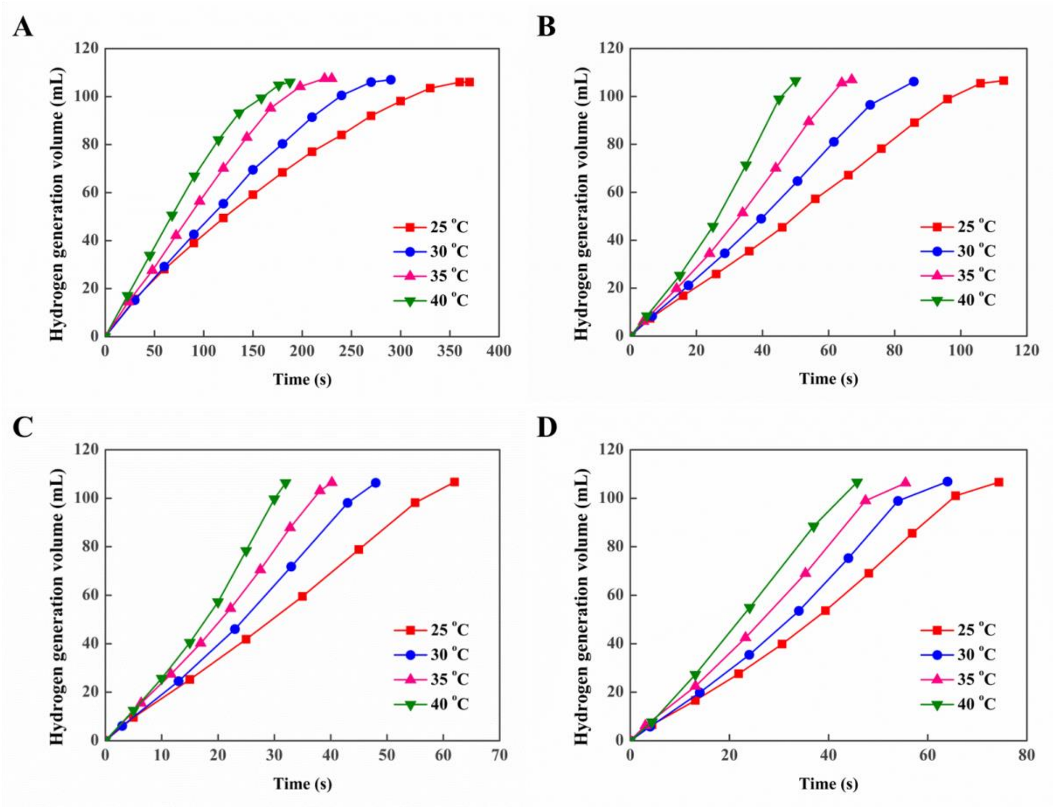

Figure S4. Catalytic activities at different reaction temperatures, related to Figure 6. Hydrogen generation as a function of time at 25, 30, 35 and 40 °C for (A) Pt/CNT, (B) Pt/CNT-1W, (C) Pt/CNT-5W and (D) Pt/CNT-10W.

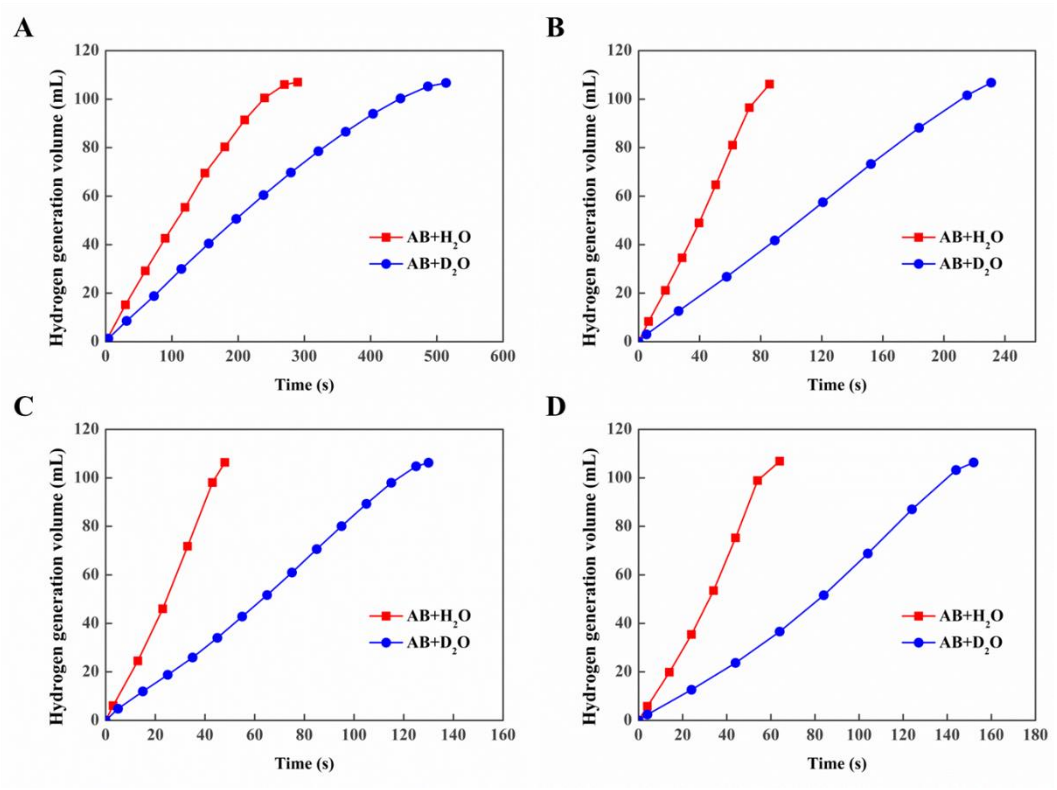

Figure S5. Kinetic isotopic results of the catalysts, related to Figure 6. Hydrogen generation as a function of time by using H_2O and D_2O as reactants at 30 ^oC for (A) Pt/CNT, (B) Pt/CNT-1W, (C) Pt/CNT-5W and (D) Pt/CNT-10W.

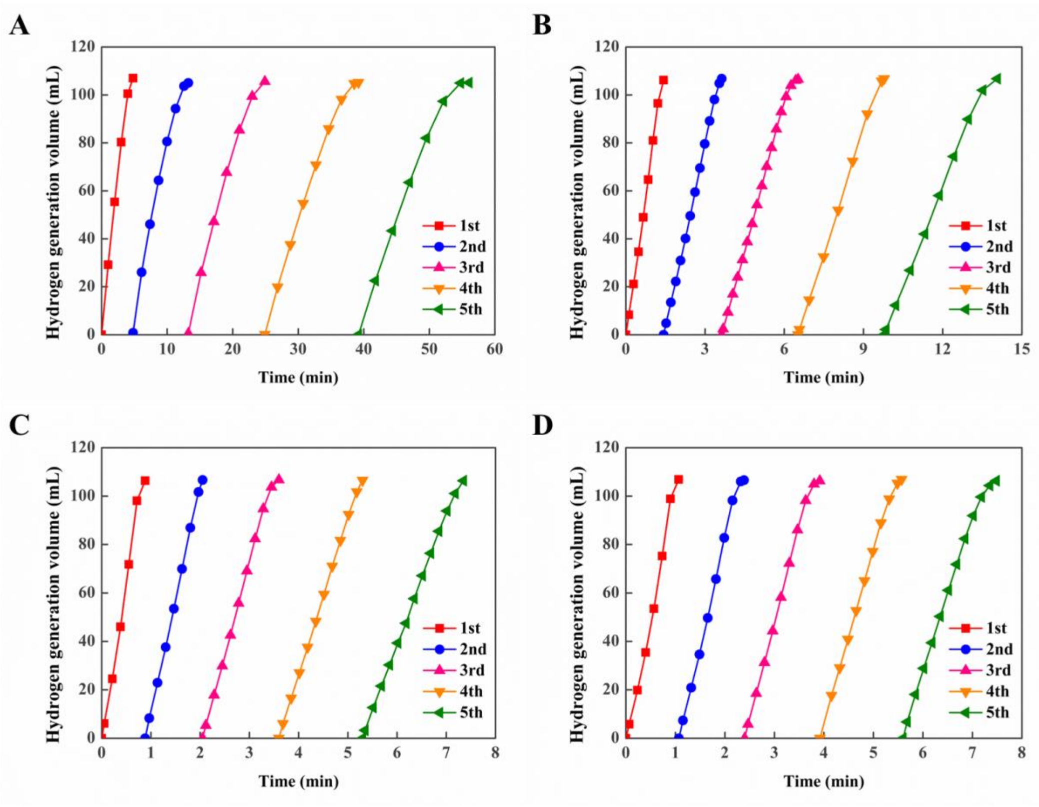

Figure S6. Catalytic durability results of the catalysts, related to Figure 6. Hydrogen generation durability at 30 °C for (A) Pt/CNT, (B) Pt/CNT-1W, (C) Pt/CNT-5W and (D) Pt/CNT-10W.

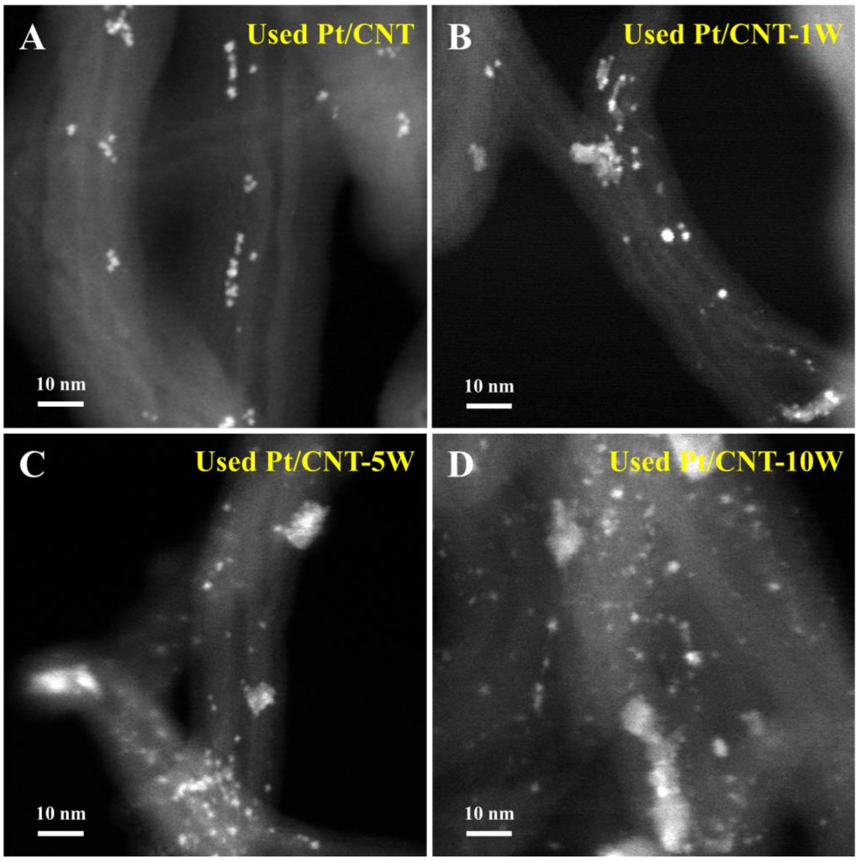

Figure S7. HAADF-STEM characterization of the used catalysts, related to Figure 6. Typical HAADF-STEM images of the used (A) Pt/CNT, (B) Pt/CNT-1W, (C) Pt/CNT-5W and (D) Pt/CNT-10W catalysts.

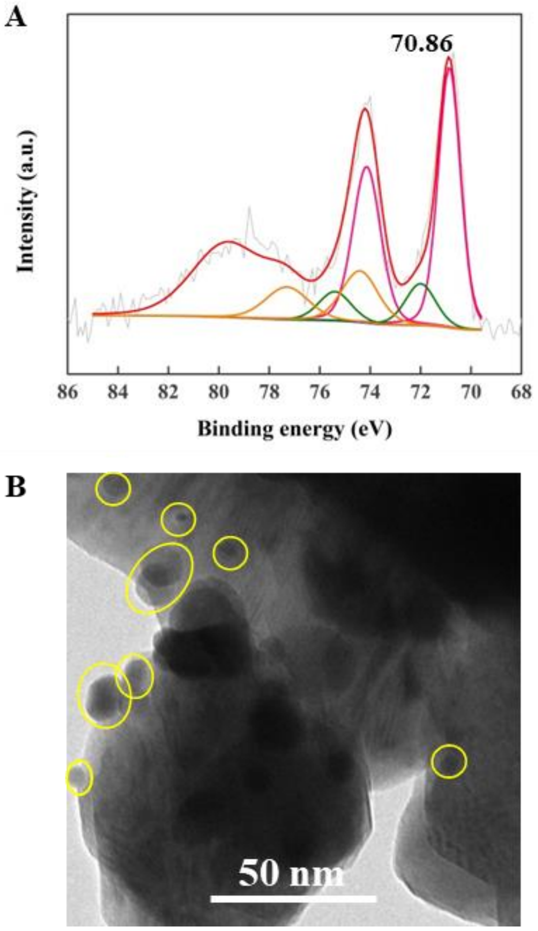

Figure S8. The electronic and structural characterization of Pt/WO_3, related to Figure 6. (A) XPS Pt 4f spectra and (B) Typical HRTEM image of Pt/WO_3.

Transparent Methods

Catalyst Preparation. Pristine multi-walled carbon nanotubes (CNT, 98%, Beijing Cnano Technology Limited) and WO_3 (99.9%, Adamas Reagent Co., Ltd.) were used as catalyst support. Ammonium tungstate hydrate ($(NH_4)_{10}W_{12}O_{41}\cdot xH_2O$, 99%, Alfa Aesar) and hexachloroplatinic acid ($H_2PtCl_6\cdot 6H_2O$, AR, Sinopharm Chemical Reagent Co. Ltd) were utilized as the metal precursors without further purification.

The tungsten-incorporated CNT-γW supported Pt catalysts were prepared by a two-step method. In the first step, the tungsten-incorporated CNT-γW hybrid materials were prepared. Typically, a given amount of ammonium tungstate hydrate was dissolved in 20 mL deionized H_2O with an electrical conductivity $<10^{-6}$ S/cm to obtain the solution with different tungsten concentrations of 1, 5 and 10 wt%. Then, 1 g pristine CNT was added into the solution under vigorous agitation, which was maintained at 90 ^{o}C for 12 h. After that, the suspension was filtered, washed with deionized H_2O for several times, and dried under stagnant air at 90 ^{o}C for 12 h. The as-obtained sample was transferred to a tubular oven and then heated from room temperature to 450 ^{o}C with a heating rate of 5 $^{o}C\cdot min^{-1}$ under Ar atmosphere, which was maintained for another 2 h. The resultant tungsten-incorporated CNT-γW was denoted as CNT-γW, which γ refers to the concentration of W in the solution. In the second step, pristine CNT, CNT-γW or WO_3 supported Pt catalysts were prepared by incipient wetness impregnation, which the supports were mixed with an aqueous solution of H_2PtCl_6 to achieve a Pt loading of 1.5 wt%. The impregnated samples were dried at room temperature and then at 80 ^{o}C under stagnant air for 12 h,

respectively. Finally, the catalyst precursors were reduced by pure H_2 with a flow rate of 40 ml·min^{-1} at 250 ^{o}C for 2 h. After the reduction, the catalysts were cooled to room temperature under Ar with a flow rate of 40 ml·min^{-1}, and then passivated by 1% O_2/Ar with a flow rate of 40 ml·min^{-1} for 20 min. The as-obtained catalysts were denoted as Pt/CNT-γW.

Characterization. X-ray diffraction (XRD) patterns of these supports were recorded using Rigaku D/Max 2550VB/PC diffractometer (Rigaku, Japan) with Cu K_α radiation. Thermogravimetric analysis (TGA) of these supports were conducted using TA SDT Q600 analyzer (TA Instruments Co., USA) from room temperature to 800 ^{o}C with a heating rate of 10 ^{o}C·min^{-1} under a constant air flow. Raman spectra were recorded using a LabRAM-HR instrument (Horiba Jobin Yvon, France) with an Ar laser (514 nm) for excitation. Hydrogen temperature programmed reduction (H_2-TPR) was carried out in an Autochem 2920 instrument (Micromeritics, USA) with a flow of 10% H_2/Ar (30 mL·min^{-1}) from room temperature to 900 ^{o}C with a heating rate of 10 ^{o}C·min^{-1}. High angle annular dark field scanning transmission electron microscopy (HAADF-STEM) imaging and energy dispersive X-ray spectroscopy (EDS) elemental mapping were performed by a F20 S-TWIN high-resolution transmission electron microscope (Tecnai G2, FEI Co. USA) with an accelerating voltage of 200 kV. High resolution transmission electron microscopy (HRTEM) observation was performed with a JSM-2100 electron microscope (JEOL, Japan). X-ray photoelectron spectra (XPS) were recorded using Kratos XSAM 800 photoelectron spectrometer

(Manchester, UK) with an Al-K_{α} X-ray source (hν=1486.6 eV). Inductively coupled plasma atomic emission spectrometer (ICP-AES) analysis was conducted using a 725-ES instrument (Agilent Technologies, USA).

Catalytic Testing. The apparatus used for catalytic activity test of ammonia borane hydrolysis was similar to that previously reported. Typically, prior to the reaction, a given amount of catalysts (0.1 g) was preloaded in a 50 mL three-necked flask containing a Teflon-coated stir bar, which was then transferred to a H_2O bath with a magnetic stirrer. The reaction was initialized by injecting an aqueous ammonia borane solution (5 mL, 0.01 g·mL^{-1}) into the reaction flask with a stirring speed of 900 rpm at 30 ^{o}C. A glass gas burette filled with H_2O was connected to the reaction flask to measure the amount of discharged H_2O, which could be weighed by an electronic balance and converted to the volume of evolved hydrogen during the reaction. The hydrolysis of ammonia borane was also conducted at various temperatures, i.e., 25, 30, 35 and 40 ^{o}C, to calculate the activation energies (E_a) of different catalysts. In order to investigate the kinetic isotope effect, the reaction was also conducted by replacing H_2O with D_2O as the reactant over different catalysts at the same reaction conditions.

In order to evaluate the durability of these catalysts, the reaction was repeated another 4 times by adding the same amount of ammonia borane solution (5 mL, 0.01 g·mL^{-1}) into the reaction flask after the completion of the last cycle. After the durability test, the used catalysts were separated from the spent solution by filtered, washed with deionized H_2O and dried under vacuum at 30 ^{o}C for characterization.

DFT Calculations. The calculations were performed with the Vienna ab initio simulation package (VASP) (Kresse and Hafner, 1993, 1994; Kresse and Furthmuller, 1996a, 1996b), implementing the spin-polarized DFT calculations in conjunction with projected augmented wave (PAW) potentials (Blochl, 1994). The exchange correlation functional was described by generalized gradient approximation of Perdew-Burke-Ernzerhof (GGA-PBE) functional (Perdew, 1996). The Kohn-Sham equation was solved using a cut off energy of 450 eV for the plane wave expansion. For Brillouin-zone integration, we used the 3 × 3 × 1 Monkhorst-Pack k-point mesh for the primitive cell (Monkhorst, 1976). The geometry optimization was converged until the residual forces of each atom were less than 0.05 eV/Å and the total energy differences less than $<10^{-7}$ eV. For Pt slab model, a p(3 × 3) supercells with four layers was under test, which the bottom two layers were fixed for the bulk optimization. For WO_3 slab model, a p(1 × 1) supercells with seven layers was under test, which the bottom four layers were fixed for the bulk optimization. The adsorption energy (E_{ads}) of species (A) on metal surface (M) is defined as $E_{ads}=E_{A/M, tot}-E_{M, tot}-E_{A, tot}$, which $E_{A/M, tot}$, $E_{M, tot}$ and $E_{A, tot}$ is the total energies of A adsorbed metal surface, clean metal surface, and A in gas phases, respectively. On the other hand, the activation energy (E_a) is defined as $E_a=E_{TS}–E_{IS}$, which E_{TS} and E_{IS} is the energy of the transition state (TS) and the most stable initial state (IS), respectively.

Supplementary References

Kresse, G.; Hafner, J. (1993). Ab initio molecular dynamics for liquid metals. *Phys. Rev. B* 47, 558-561.

Kresse, G.; Hafner, J. (1994) Ab initio molecular-dynamics simulation of the liquid-metal-amorphous-semiconductor transition in germanium. *Phys. Rev. B* 49, 14251-14269.

Kresse, G.; Furthmuller, J. (1996) Efficiency of ab-initio total energy calculations for metals and semiconductors using a plane-wave basis set. *Comp. Mater. Sci.* 6, 15-50.

Kresse, G.; Furthmuller, J. (1996) Efficient iterative schemes for ab initio total-energy calculations using a plane-wave basis set. *Phys. Rev. B* 54, 11169-11186.

Blochl, P. E. (1994) Projector augmented-wave method. *Phys. Rev. B* 50, 17953-17979.

Perdew, J. P.; Burke K.; Ernzerhof M. (1996) Generalized gradient approximation made simple. *Phys. Rev. Lett.* 77, 3865-3868.

Monkhorst, H. J.; Pack, J. D. (1976) Special points for Brillouin-zone integrations. *Phys. Rev. B* 13, 5188-5192.
